# Protective Effect of Geranylgeranylacetone via Enhancement of HSPB8 Induction in Desmin-Related Cardiomyopathy

**DOI:** 10.1371/journal.pone.0005351

**Published:** 2009-04-28

**Authors:** Atsushi Sanbe, Takuya Daicho, Reiko Mizutani, Toshiya Endo, Noriko Miyauchi, Junji Yamauchi, Kouichi Tanonaka, Charles Glabe, Akito Tanoue

**Affiliations:** 1 Department of Pharmacology, National Research Institute for Child Health and Development, Tokyo, Japan; 2 Department of Pharmacology, Tokyo University of Pharmacy and Life Science, Tokyo, Japan; 3 Department of Molecular Biology and Biochemistry, University of California Irvine, Irvine, California, United States of America; Universidad Peruana Cayetano Heredia, Peru

## Abstract

**Background:**

An arg120gly (R120G) missense mutation in HSPB5 (α-β-crystallin ), which belongs to the small heat shock protein (HSP) family, causes desmin-related cardiomyopathy (DRM), a muscle disease that is characterized by the formation of inclusion bodies, which can contain pre-amyloid oligomer intermediates (amyloid oligomer). While we have shown that small HSPs can directly interrupt amyloid oligomer formation, the *in vivo* protective effects of the small HSPs on the development of DRM is still uncertain.

**Methodology/Principal Findings:**

In order to extend the previous *in vitro* findings to *in vivo*, we used geranylgeranylacetone (GGA), a potent HSP inducer. Oral administration of GGA resulted not only in up-regulation of the expression level of HSPB8 and HSPB1 in the heart of HSPB5 R120G transgenic (R120G TG) mice, but also reduced amyloid oligomer levels and aggregates. Furthermore, R120G TG mice treated with GGA exhibited decreased heart size and less interstitial fibrosis, as well as improved cardiac function and survival compared to untreated R120G TG mice. To address possible mechanism(s) for these beneficial effects, cardiac-specific transgenic mice expressing HSPB8 were generated. Overexpression of HSPB8 led to a reduction in amyloid oligomer and aggregate formation, resulting in improved cardiac function and survival. Treatment with GGA as well as the overexpression of HSPB8 also inhibited cytochrome c release from mitochondria, activation of caspase-3 and TUNEL-positive cardiomyocyte death in the R120G TG mice.

**Conclusions/Significance:**

Expression of small HSPs such as HSPB8 and HSPB1 by GGA may be a new therapeutic strategy for patients with DRM.

## Introduction

Many systemic and neurodegenerative disorders whose etiologies are linked to misfolded or unfolded proteins are characterized by the accumulation of intracellular or extracellular protein deposits or aggregates known as aggresomes [1], [2]. As well as neurodegenerative pathologies and other age-related illnesses, many recent publications suggest that the accumulation of misfolded or unfolded proteins contributes to a number of cardiovascular diseases such as cardiomyopathy, myocardial infarction and heart failure [3], [4], [5]. Heat shock proteins (HSPs) are present to prevent the accumulation of the misfolded or unfolded proteins in the cells. HSPs can protect other proteins as molecular chaperones against misfolding by stabilizing them, and usually remove them when misfolded [6]. Lower levels of HSPs, or reduced efficacy of molecular chaperones could be associated with cardiovascular disease and aspects of ageing [7], [8]. It is also known that mutations or other changes in the chaperone proteins themselves cause them to actually promote misfolding, rather than guard against it [9]. This implies that cellular levels of HSPs play a critical role in progression and severity of disease linked to misfolded or unfolded proteins.

The small HSPs, such as HSPB5 (alpha-beta-crystallin), HSPB8 (HSP22 or H11 kinase) and HSPB1 (HSP25), share sequence similarity within the α-crystallin domain but exhibit different patterns of gene expression, transcriptional regulation, and sub-cellular localization [10]. The chaperone-like activity of small HSPs, which are classified according to their ability to prevent protein aggregation and/or restore the biological activity of the substrates, is widely believed to be a protective mechanism for protein misfolding and denaturation triggered by noxious environmental stimuli, such as hyperthermic stress, heavy metals, ischemic injury, and some genetic diseases [2], [10], [11].

The arg120gly (R120G) missense mutation in HSPB5 can cause desmin-related cardiomyopathy (DRM) [12]. This disease, which is characterized by the formation of aggregates containing HSPB5 and desmin, is a misfolded protein-related disease and can be recapitulated in transgenic (TG) mice by expressing the mutant HSPB5 R120G protein specifically in the heart [9]. We showed that HSPB5 R120G caused perinuclear aggresome formation and that these aggresomes contained pre-amyloid oligomer intermediates (amyloid oligomer) [3]. These results suggest that DRM caused by the HSPB5 mutation is a subclass of aggresomal and amyloid-related diseases [3]. Furthermore, using a cardiac-specific inducible TG system [13], we found that continuous expression of the mutant protein is necessary for sustaining the high concentrations of the amyloid oligomer that correlate with depressed cardiac function as well as premature lethality [14]. These results indicate that HSPB5-DRM is at least partially reversible and that the amyloid oligomer is critical for aspects of morbidity and mortality characteristic of DRM [14].

In our previous study, we showed that recombinant HSPB8 or HSPB1 protein could directly interrupt HSPB5 R120G-mediated amyloid oligomer formation although amyloid oligomer is still present in the mixture of wild-type (WT) HSPB5 and mutant HSPB5 R120G [15]. This interruption by HSPB1 and HSPB8 was confirmed in a cardiomyocyte-based study using an adenoviral vector system. Blockade of amyloid oligomer formation by HSPB1 and HSPB8 led to the recovery of ubiquitin proteasomal activity and cellular viability. These results imply that blockade of the amyloid oligomer formation by small HSPs may be a new therapeutic strategy for treating patients with DRM as well as other types of amyloid-based degenerative diseases. While we have shown that HSPB1 and HSPB8 can directly interrupt amyloid oligomer formation caused by HSPB5 R120G and restore cellular viability in cardiomyocytes expressing HSPB5 R120G, the *in vivo* protective effects of the small HSPs on the development of DRM remain unproven.

In the present study, we show that geranylgeranylacetone (GGA), a nontoxic antiulcer drug and inducer of small HSPs [16], can induce expression of HSPB8 and HSPB1 and reduce the formation of amyloid oligomers as well as insoluble aggregates in HSPB5 R120G TG mice. GGA led to reduction in heart size and inhibition of interstitial fibrosis, and recovery of cardiac function as well as improved survival. Cardiac-specific TG mice expressing HSPB8 also inhibit the progression of cardiomyopathy in the R120G TG mice, suggesting that the induction of HSPB8 may play a role in inhibiting the development of DRM. Together, these results indicate that induction of small HSPs is beneficial in the treatment of DRM.

## Materials and Methods

### Trangenic mice

Male mice with cardiac-specific overexpression of wild-type HSPB5 (WT TG) or mutant HSPB5 containing the R120G mutation (R120G TG), driven by the α-myosin heavy chain promoter, have been described [3]. To generate mice with cardiac-specific inducible overexpression of HSPB8, HSPB8 cDNA was inserted into a modified myosin promoter cassette as described previously[13], [15] and generated HSPB8 TG mice. The responder HSPB8 mice were crossed with the tetracycline-controlled transcriptional activator (tTA) TG mice to generate the tTA/HSPB8 double TG (tTA/HSPB8 TG) mice [13], [14]. The TG mice were identified by PCR analysis of genomic DNA isolated from tail tips. The genetic background of the responder HSPB8 mice used for all experiments was a C57BL/6Cr Slc (SLC, Shizuoka, Japan). The TG mice such as WT TG, R120G TG and tTA TG mice was backcrossed with a C57BL/6Cr Slc mouse more than 8 times, and maintained on a C57BL/6Cr Slc background. Animals were housed in microisolator cages in a pathogen-free barrier facility. All experimentation was performed under approved institutional guidelines. Non-transgenic (NTG) littermates animals with the same genetic background were always used as controls for comparison. Animals were housed in microisolator cages in a pathogen-free barrier facility. All experiments were performed under the approved guidelines for the Care and Use of Laboratory Animals of the National Research Institute for Chile Health and Development.

### Cardiomyocyte cultures and adenovirus infection

After isolation of rat neonatal cardiomyocytes, cells were grown on glass slides coated with a gelatin as described previously [3], [15]. Replication-deficient recombinant adenoviruses were made using an AdEasy system [3], [15]. The cells were normally infected at a multiplicity of infection of 10 for each virus except where indicated.

### Experimental design and protocol for drug treatment in *in vitro* and *in vivo*

GGA was purchased from Eisai, Inc. (Tokyo, Japan). For the study with cardiomyocytes, GGA was dissolved in ethanol, and ethanol alone was used as the vehicle (0 nM of GGA). After 24-hr isolation of the cardiomyocytes, GGA was added into the culture medium and sustained until the end of the experiments. Twenty-four hr after GGA treatment, an adenoviral vector containing HSPB5 R120G was infected into the cardiomyocytes. Forty-eight hours after infection, Western blotting, cellular viability assay and immunohistochemistry were performed.

For oral administration in mice, the GGA granules were mixed with powdered rodent chow at concentrations of 0.25%, 0.5%, 1%, and 2%, and each powdered rodent chow containing GGA was given to the mice. All mice had unlimited access to food and water. After TG mice were genotyped by analysis of their tail tips, HSPB5 R120G TG mice were given GGA starting at 4 weeks of age and continuing for 41 weeks. By the time GGA was started, obvious aggresomes containing HSPB5 were already visible in the HSPB5 R120G TG mouse hearts [9]. Thus, we started administering GGA at an early stage of disease in the DRM. Western blotting, morphological analysis, immunohistochemistry and echocardiography were performed on the mice at 30 weeks of age as described above.

To evaluate the effect of HSPB8 on the development of the cardiac disease caused by the HSPB5 R120G mutation, HSPB5 R120G TG mice were crossbred with tTA/HSPB8 double transgenic (tTA/HSPB8 TG) mice to generate tTA/HSPB8/R120G triple transgenic mice (Triple TG). Multiple analyses such as Western blotting, morphology and echocardiography were performed on NTG, R120G TG, tTA/HSPB8 TG, and triple TG mice at 30 weeks of age. In some sets of experiments, we analyzed the triple TG mice treated with doxycycline as described previously [13], [14]. In these cases, doxycycline treatment was started in the triple TG mice at 4 weeks of age and continued until 30 weeks of age.

### Immunohistochemistry

Immunohistochemical analyses were performed as described [3], [14], [15]. Alexa488-conjugated anti-rabbit, Alexa568-conjugated anti-mouse antibodies, and phalloidin-Alexa 488 were from Molecular Probes (Eugene, OR). Anti-HSPB5 antibody (SPA-223) was from Assay Designs, Inc. (Ann Arbor, MI), anti-troponin I (TnI) antibody (MAB1691) was from Chemicon International (Temecula, CA) and anti-HSPB8 antibody was from Imgenex (San Diego, CA). The anti-oligomer antibody (A-11) was generated and used as described previously [3]. The cellular viability was measured using a 3-(4,5-dimethylthiazol-2-yl)-2,5-diphenyl tetrazolium bromide (MTT) assay [15]. Image J 1.36b was used to quantify the immunofluorescent intensity. The results from 30–50 cells were averaged for cohort comparison. The area stained with the oligomer antibody was defined, and the average pixel intensity of the cardiomyocyte was determined for comparison [15].

### Miscellaneous methods

Sample preparation for Western blotting, gel preparation, and electrophoretic conditions have been described [14], [17], [18]. Western blot analyses were performed with the use of anti-glyceraldehyde-3-phosphate dehydrogenase (GAPDH) (Chemicon International, Temecula, CA), anti-HSP70 (Ab-1, Merck Co. Inc., Whitehouse Station, NJ), anti-HSP B5 antibody (SPA-223, Assay Designs Inc., Ann Arbor, MI), anti-HSP B1 (SPA-801, Assay Designs Inc., Ann Arbor, MI), anti-HSP B8 antibody (Imgenex, San Diego, CA), anti-heat shock factor (HSF)1(SPA-901, Assay Designs Inc., Ann Arbor, MI), anti-Cytochrome C (BD Bioscience, CA), anti-voltage dependent anion channel (VDAC) (EMD Chemicals Inc., Gibbstown, NJ), and anti-activated caspase-3 (Abcam Inc., Cambridge, MA). The band intensity in the immunoblot was semi-quantified using Image J1.36b. The filter assay for the detection of the aggregates was performed as described previously [3], [14]. The aggregate fraction on the membrane was detected with an anti-HSP B5 antibody. TdT-mediated dUTP-biotin Nick End Labeling (TUNEL) staining was carried out using an *in situ* cell death detection kit, TMR Red (Roche, Basel, Switzerland), following the manufacturer's instructions as described previously [18]. The number of DAPI-labeled nuclei was counted and compared to the number of TUNEL-positive cells [18]. Trichrome staining was performed as described [17]. Trichrome staining was performed as described [17].

### Echocardiography

Echocardiography was performed as described previously [19]. Mice were anesthetized with an intraperitoneal injection of 30 mg/kg pentobarbital (Sigma, St. Louis, MO). After anesthesia, the left hemithorax of the mouse was shaved. The animals were pre-warmed with a panel heater to maintain their rectal temperature at 37°C during the determination of cardiac parameters by echocardiography. Transthoracic echocardiography was performed by using ProSound 5500R (Aloka, Tokyo, Japan) with a 13-MHz linear transducer for mice in a phased array format, which offers 0.35-mm lateral resolution and 0.25-mm axial resolution, real time digital acquisition, storage, and review capabilities. The heart was first imaged in the two-dimensional mode in the parasternal long-axis view. From this view, an M-mode cursor was positioned perpendicular to the interventricular septum and posterior wall of the left ventricle at the level of the papillary muscles. Chamber dimensions were determined by the M-mode tracings. In contrast, a two-dimensional short-axis view of the mid-left ventricle at the chordal level was assessed by the B-mode tracings. Wall thickness was determined from this dimensional short-axis view. Each cardiac parameters was calculated from the echocardiogram as described previously [19].

### Statistics

Data are expressed as the mean±standard error. Statistical analysis was performed using the unpaired Student's t-test and one-way or two-way ANOVA followed by a post hoc comparison with Fisher's PLSD using Statview version 5.0 software (Concepts, Inc., Berkeley, CA, USA). Differences between groups were considered statistically significant at the level of *p*<0.05.

## Results

### GGA induces small HSPs in cardiomyocytes

**Figure 1:**
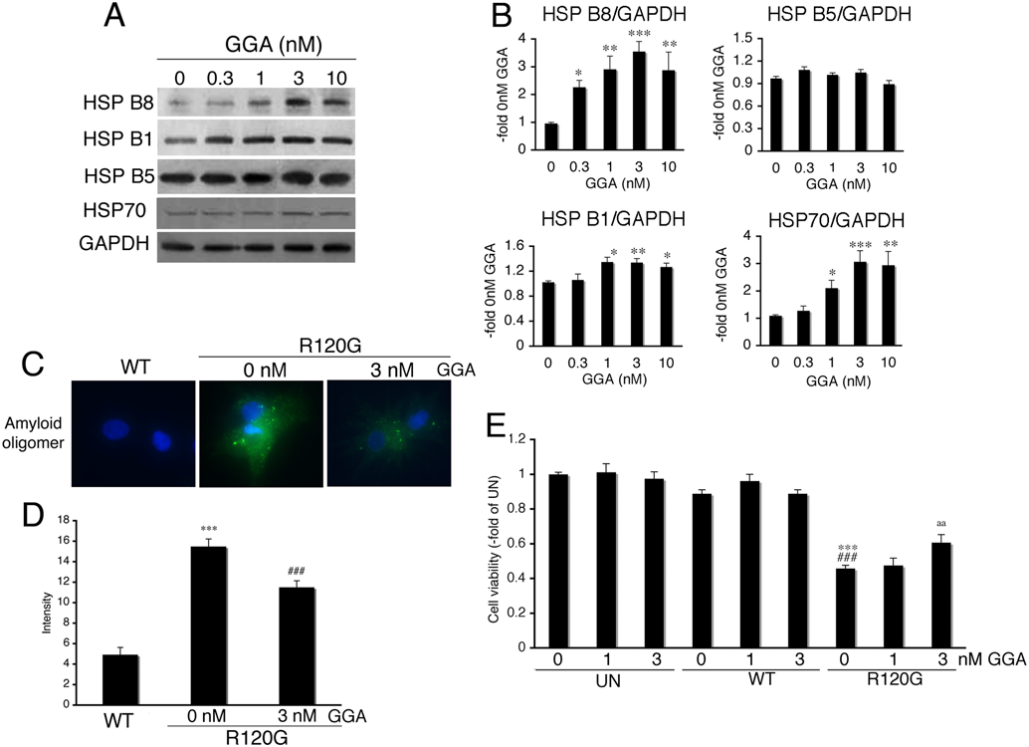
Induction of small HSPs by GGA in neonatal rat cardiomyocytes. (A) Western blot analysis. GGA induced the expression levels of small HSPs in a dose-dependent manner. (B) Quantitative analysis of small HSP expression (n = 4). Values are the -fold increase relative to cardiomyocyte cultures treated with vehicle (0 nM of GGA) whose values are arbitrarily set to 1. * *p*<0.05, ** *p*<0.01 and *** *p*<0.001 vs. 0 nM of GGA. (C) Representative pictures of immunohistochemical analyses in the HSPB5 R120G-infected cardiomyocytes. Cardiomyocytes were infected with the adenovirus vector containing the wild-type HSPB5 (WT) or mutant HSPB5 R120G (R120G). An amyloid oligomer (green) was detected by the anti-oligomer antibody as described in Materials and Methods. Nuclei were stained with DAPI. (D) Quantitative analysis of the amyloid oligomer. Amyloid oligomer levels were measured by fluorescence intensity (n = 4). *** *p*<0.001 vs. cardiomyocytes infected with WT, and ### *p*<0.001 vs. cardiomyocytes infected with R120G. (E) Protective effects of GGA on the cellular viability of the HSPB5 R120G-infected cardiomyocytes. Cellular viability was determined by the MTT assay. Values are the -fold increase relative to untreated cardiomyocyte cultures whose values are arbitrarily set to 1 (n = 6). *** *p*<0.001 vs. untreated cardiomyocytes (UN), ### *p*<0.001 vs. cardiomyocytes infected with WT, and aa *p*<0.01 vs. cardiomyocytes infected with R120G.

Effects of GGA on the expression level of small HSPs were evaluated in the isolated neonatal rat cardiomyocytes. While GGA increased the expression levels of HSPB8 and HSPB1 as well as HSP70 in a dose-dependent manner, no induction of HSPB5 was observed (Figure 1A and B). These results suggest that GGA can induce some stress proteins in the cardiomyocytes [16], [20]. Since we have found that HSPB8 and HSPB1 can reduce the amyloid oligomer toxicity generated by HSPB5 R120G [15], we analyzed the effect of GGA on generating amyloid oligomers caused by HSPB5 R120G expression (Figure 1C and D). As expected, amyloid oligomer levels increased (Figure 1C and D), reducing viability (Figure 1E). GGA treatment partially inhibited the increase in amyloid oligomer formation and increased viability (Figure 1C, D and E), suggesting that GGA is protective against the cellular toxicity induced by HSPB5 R120G.

### Effect of GGA on stress protein levels in HSPB5 R120G TG mouse hearts

**Figure 2:**
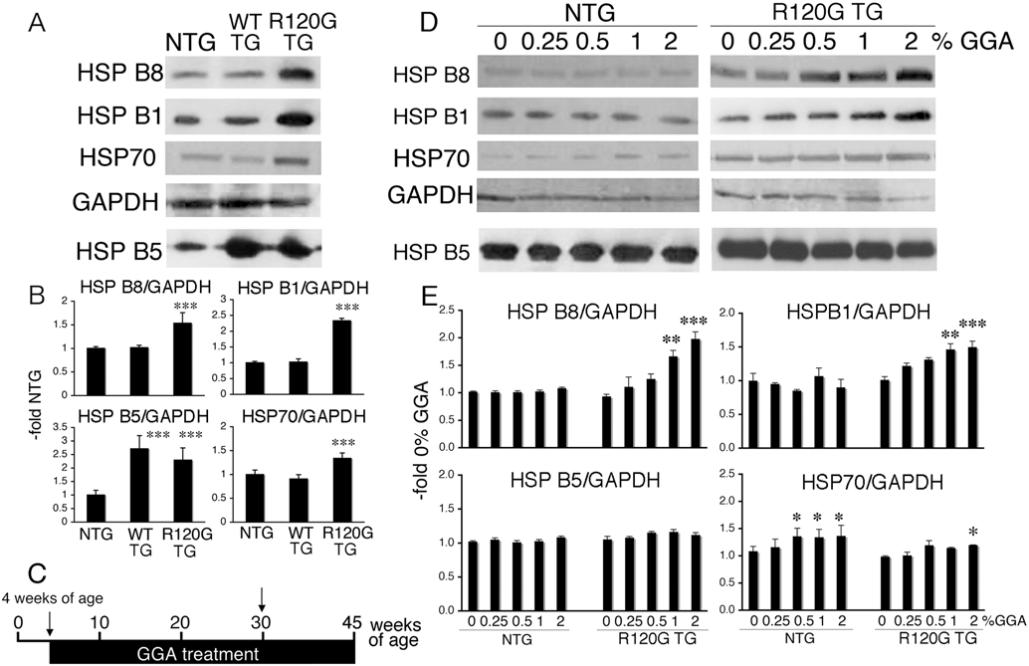
In vivo enhancement of small HSP induction by GGA. (A) Representative pictures of Western blot analysis of the R120G TG mouse heart. (B) Quantitative analysis of the small HSP expression levels (n = 4 mice). Values are the -fold increase relative to those in NTG mice whose values are arbitrarily set to 1. *** *p*<0.001 vs. NTG. Values of wild-type HSPB5 TG mice (WT TG) are also shown as a control. (C) Diagram of the protocols. NTG and HSPB5 R120G TG mice at 4 weeks of age were administered GGA. Western blotting, morphological analysis, immunohistochemistry and echocardiography were performed on the mice at 30 weeks of age (arrow). (D) Representative pictures of Western blot analysis of hearts from R120G TG mice treated with GGA. GGA was administrated in R120G TG mice from 4 weeks until 30 weeks of age. (E) Quantitative analysis of the small HSP expression levels (n = 4–5 mice). Values are the -fold increase relative to those in NTG mice whose values are arbitrarily set to 1. * *p*<0.05, ** *p*<0.01 and *** *p*<0.001 vs. 0% GGA.

In order to extend these in vitro data, the in vivo effects of GGA were examined in the mouse hearts. We first analyzed expression levels of small HSPs in the hearts from R120G TG mice at 30 weeks of age (Figure 2A and B). The expression level of HSPB5 was similar between R120G TG and WT TG mice as described previously [9]. Although the expression levels of HSPB5 in the WT and R120G hearts were similar, HSPB8, HSPB1 and HSP70 were up-regulated in the hearts from R120G TG mice as compared with those of HSPB5 WT TG and NTG mice (Figure 2A and B). There were no differences in HSPB8, HSPB1 and HSP70 levels between WT TG and NTG mice, although HSPB5 was overexpressed in WT TG mice (Figure 2A and B). Up-regulation of the small HSPs is probably due to a cellular adaptation in response to stress caused by the mutant HSPB5 R120G protein [3]. We then examined the long-term effects of GGA on small HSP expression in the NTG and HSPB5 R120G TG mice (Figure 2C–E). HSPB5 R120G TG mice were given GGA starting at 4 weeks of age and continuing for 41 weeks (Figure 2C). Western blotting, morphological analysis, immunohistochemistry and echocardiography were performed on the mice at 30 weeks of age as described in Materials and Methods (Figure 2C, arrow). HSP70 was significantly increased in NTG mice by oral administration of GGA (Figure 2D and E), whereas no obvious induction of small HSPs such as HSPB8, HSPB1 or HSPB5 was observed. In contrast to NTG mice, GGA treatment markedly enhanced the induction of HSPB8 and HSPB1 in R120G TG mice (Figure 2D and E) with a slight induction of HSP70 (Figure 2D and E). These results suggest that GGA can profoundly enhance the induction of a discrete subset of small HSPs in R120G TG mouse hearts.

### Effect of GGA on HSPB5 R120G cardiomyopathy

**Figure 3:**
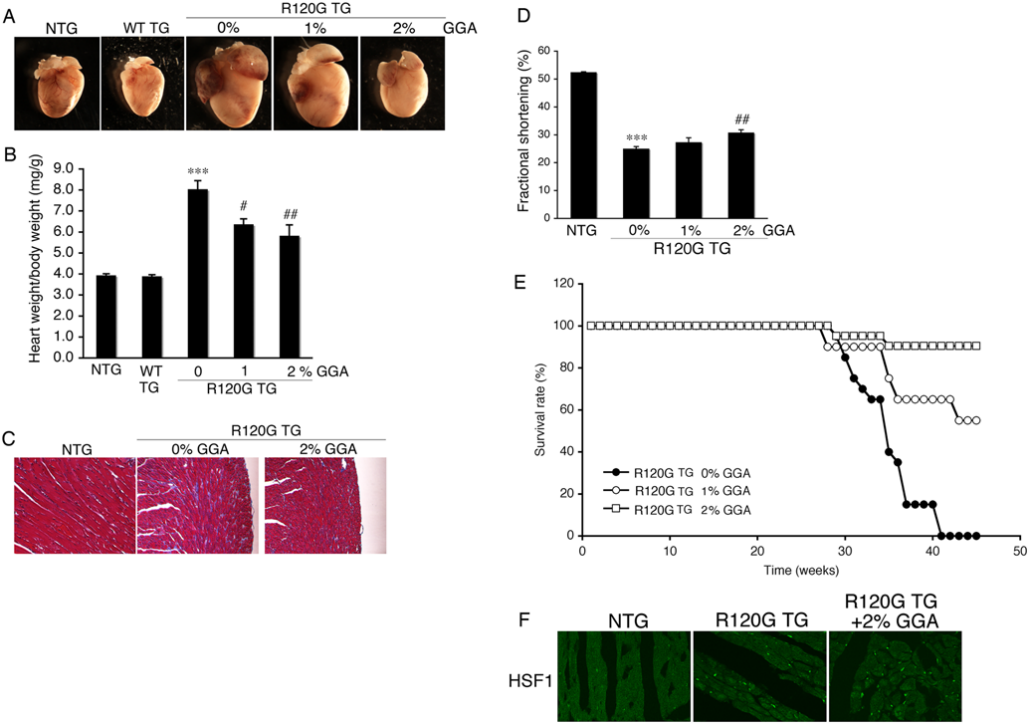
Effect of long-term GGA treatment on development of cardiomyopathy in HSPB5 R120G TG (R120G TG) mice. (A) Representative pictures of the hearts in the R120G TG mice at 30 weeks of age. (B) The ratios of heart weight per body weight. Long-term GGA treatment inhibited hypertrophy in R120G TG mice (n = 6–8 mice). (C) Masson's trichrome stain in the R120G TG mouse heart with or without GGA treatment (2% GGA). (D) Fractional shortening. Cardiac functional measurement was carried out at 30 weeks of age (n = 10–15 mice). (E) Survival curves. GGA treatment effectively attenuated premature death in the R120G TG mice (n = 15–20 mice). (F) Heat shock factor (HSF1) in the hearts. Localization of HSF1 is shown in heart from R120G TG mice. ***p<0.001 vs. NTG mice and # *p*<0.05, ## *p*<0.01, and ### *p*<0.001 vs. R120G TG mice with 0% GGA (0% GGA in the R120G TG mice).

**Figure 4:**
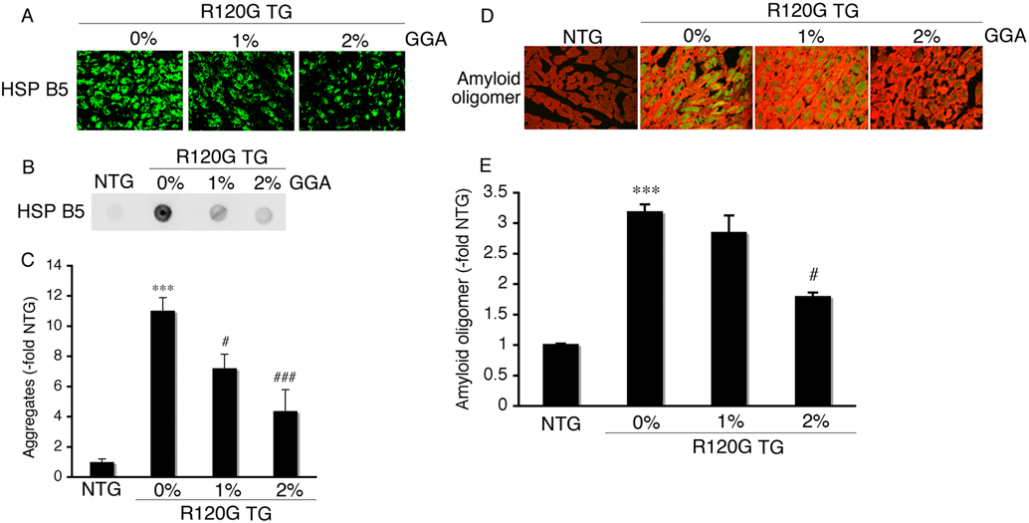
Inhibition of aggregate (A–C) and amyloid oligomer formation (D–E) by GGA treatment in HSPB5 R120G TG (R120G TG) mice at 30 weeks of age. (A) Representative pictures of the immunohistochemistry are shown. The aggregates containing the mutant HSPB5 R120G protein were observed in the R120G TG mice, and long-term GGA treatment reduced the aggregates. (B) Typical picture of the filter assay for the detection of the aggregates. (C) Quantitative analysis of the aggregates containing the mutant HSPB5 R120G protein (n = 4 mice). (D) Effect of GGA on expression level of the amyloid oligomer. The amyloid oligomer (green) was stained by the anti-oligomer antibody as described in the Materials and Methods section. To distinguish the cardiomyocytes, cardiac troponin I was stained (red). (E) Quantitative analysis of the amyloid oligomer. Amyloid oligomer levels were measured by fluorescence intensity. GGA treatment reduced the HSPB5 R120G-induced amyloid oligomer formation. Values shown are the -fold increase relative to NTG mice, whose value was set to 1. (n = 4 mice) *** *p*<0.001 vs. NTG mice; # *p*<0.05, ### *p*<0.001 vs. R120G TG mice.

Small HSPs, such as HSPB1 and HSPB8, could be protective in HSPB5 R120G induced-cellular toxicity [15]. Therefore, we analyzed hearts and cardiac function with or without GGA treatment. The heart size in HSPB5 R120G TG were greatly enlarged relative to NTG (Figure 3A and B). Concomitant with hypertrophy, interstitial fibrosis and reduced cardiac function were also observed (Figure 3C–E, Table 1). These results imply that R120G TG mice can recapitulate the DRM pathology, resulting in eventual heart failure and death as described previously [3], [14]. GGA treatment attenuated the development of cardiac hypertrophy in R120G TG mice in a dose-dependent manner (Figure 3A and B). Partial improvements in cardiac function and inhibition of interstitial fibrosis were also observed (Figure 3C–D, Table 1) as well as a significant improvement in probability of survival over time (Figure 3E). Since it is known that protein interaction between heat shock factor (HSF) and HSP70, as well as nuclear translocation of HSF is important for HSP induction by GGA [20], the effect of GGA on HSF1 localization was examined (Figure 3F). While HSF1 was present in the cytosol of the NTG hearts, obvious nuclear localization was seen in the R120G TG hearts with or without GGA treatment (Figure 3F). GGA treatment decreased aggregate accumulation as well as amyloid oligomer formation in the R120G TG hearts (Figure 4A–E). These results indicate that long-term GGA treatment can suppress development of HSPB5 R120G-induced cardiac disease.

**Table 1:** Echocardiographic parameters.

	Body weight (g)	LVIDd (mm)	LVIDs (mm)	LVESV (µl)	LVEDV (µl)	Ejection Fraction (%)
NTG	27±2	3.2±0.2	1.5±0.1	3.8±0.6	35±6	89±3
R120G TG+0%GGA	29±2	4.3±0.1*	3.3±0.1*	35.8±3.0*	82±4*	57±2*
R120G TG+1%GGA	27±2	4.0±1.0	2.9±0.1	26.7±2.9#	66±5#	61±3
R120G TG+2%GGA	30±2	3.8±1.0#	2.7±0.1#	20.5±2.3#	59±4#	66±2#
NTG	32±2	3.5±0.2	1.9±0.1	6.6±0.4	45±6	85±2
tTA/HSP B8 TG	30±2	3.5±0.2	1.8±0.2	6.8±1.6	45±7	86±2
R120 G TG	30±2	4.3±0.1*	3.2±0.1*	35.4±3.2*	82±4*	57±2*
Triple TG	32±2	4.0±0.1#	2.8±0.1#	23±2#	64±3#	65±2#

Values are mean±SEM. N = 10–15 in each group.

LVIDd indicates left ventricular internal end-diastolic dimension; LVIDs, left ventricular internal end-systolic dimension; LVESV, left ventricular end-systolic volume; LVEDV, left ventricular end-systolic volume.

* p<0.05 vs. NTG.

# p<0.05 vs. R120G TG.

### HSPB8 can suppress HSPB5 R120G cardiomyopathy in vivo

**Figure 5:**
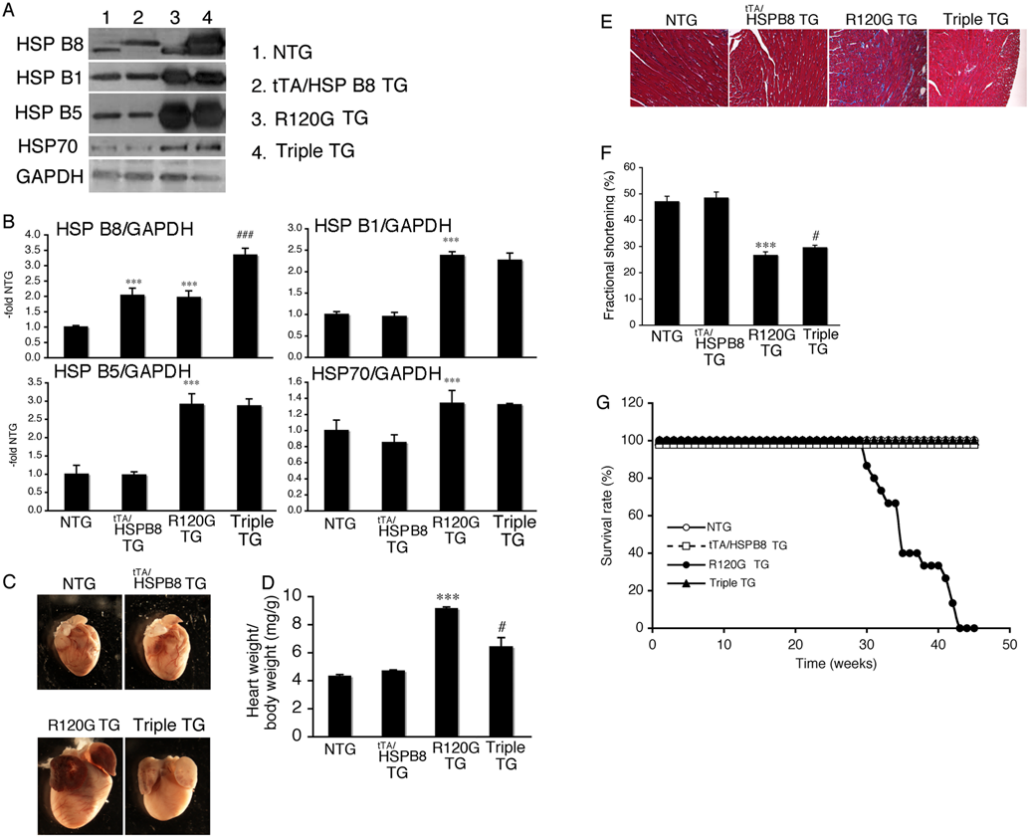
HSPB8 was overexpressed using tTA/inducible α-myosin heavy chain promoter system as described in Materials and Methods. (A) Representative Western blots using hearts from tTA/HSPB8 double transgenic (tTA/HSPB8 TG) mice, HSPB5 R120G transgenic (R120G TG) and tTA/HSPB8/HSPB5 R120G triple transgenic (Triple TG) mice. (B) Quantitative analysis of small HSP levels (n = 4 mice). Values are the -fold increase relative to those in non-transgenic (NTG) mice whose values are arbitrarily set to 1. (C) Representative pictures of R120G TG, tTA/HSPB8 TG and Triple TG hearts at 30 weeks of age. (D) The ratios of heart weight per body weight. Overexpression of HSPB8 inhibited cardiac enlargement in R120G TG mice (n = 6–8 mice). (E) Masson's trichrome stain. Overexpression of HSPB8 attenuated the interstitial fibrosis in R120G TG mice. (F) Fractional shortening assessed by the echocardiogram. Cardiac functional measurements were made at 30 weeks (n = 10–15 mice). (G) Survival curves. Overexpression of HSPB8 effectively attenuated premature death in R120G TG mice (n = 15–20 mice). *** *p*<0.001 vs. NTG mice and # *p*<0.05, and ### *p*<0.0001 vs. the R120G TG.

**Figure 6:**
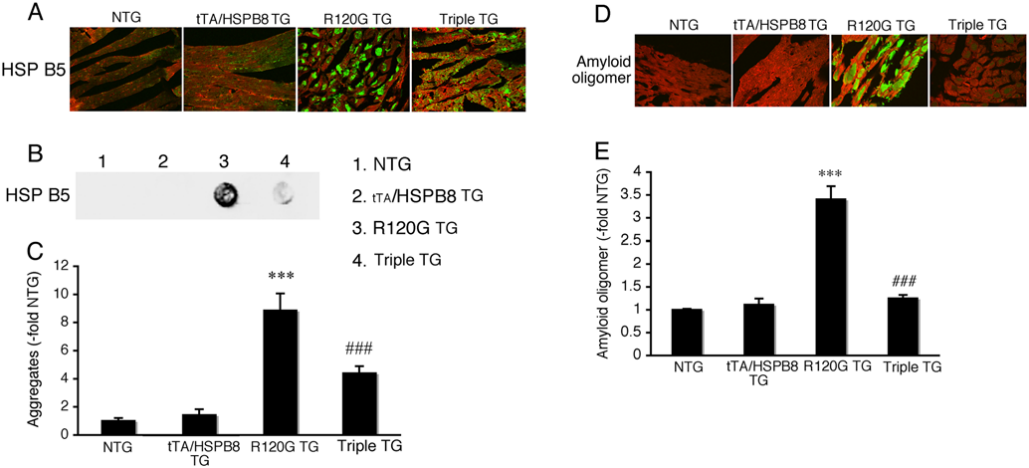
Inhibition of aggregate formation (A–C) and amyloid oligomer formation (D–E) by overexpression of HSPB8 in HSPB5 R120G TG mice (R120G TG) at 30 weeks of age. (A) Representative pictures of the immunohistochemistry are shown. The aggregates containing the mutant HSPB5 R120G protein are observed in R120G TG mice, and the overexpression of HSPB8 reduced the aggregates in samples from HSPB8 TG mice (tTA/HSPB8/HSPB5 R120G triple TG; Triple TG). (B) Typical picture of the filter assay for the detection of the aggregates. (C) Quantitative analysis of the aggregates containing mutant HSPB5 R120G protein (n = 4 mice). (D) Effect of HSPB8 overexpression on amyloid oligomer formation (green). (E) Amyloid oligomer levels were measured by fluorescence intensity. The overexpression of HSPB8 reduced the HSPB5 R120G-induced amyloid oligomer formation (Triple TG). Values shown are the -fold increase relative to NTG mice, whose value was set to 1 (n = 4 mice). To distinguish the cardiomyocytes, cardiac troponin I was stained (red)(A and D). *** *p*<0.001 vs. NTG mice; ### *p*<0.001 vs. R120G TG mice.

To clarify the functional role of HSPB8 induction on the development of HSPB5 R120G cardiomyopathy and test sufficiency, we generated a TG mouse, in which HSPB8 is overexpressed in a cardiac-specific manner using the inducible α-myosin heavy chain promoter [13] (Figure 5). The expression level of HSPB8 in tTA/HSPB8 double TG (tTA/HSPB8 TG) mice was twice the NTG level, while expression of the other HSPs tested was unchanged (Figure 5A and B). No significant differences in cardiac function, heart weight, histology or survival rate could be detected in these mice, suggesting that twice the level of overexpression of HSPB8 is nontoxic for cardiomyocytes (Figure 5A–G, Table 1). Next, we crossbred HSPB8 TG mice with R120G TG mice and generated tTA/HSPB8/R120G triple TG mice, which overexpress HSPB5 R120G and HSPB8 proteins in a cardiac-specific manner. The expression level of HSPB8 in hearts of R120G TG and tTA/HSPB8/R120G triple TG mice was 1.9- and 3.4-fold that of NTG mice, respectively, while the other small HSPs including HSPB5 were similar between the tTA/HSPB8/R120G triple TG mice and the R120G TG mice (Figure 5A and B). The tTA/HSPB8/R120G triple TG mice showed decreased heart weight, improved cardiac function and reduced interstitial fibrosis as compared with those of R120G TG mice (Figure 5C–F, Table 1). Premature death was completely suppressed by the overexpression of HSPB8 in R120G TG mice (Figure 5G). Aggregate accumulation as well as amyloid oligomer level was reduced as well (Figure 6A–E).

**Figure 7:**
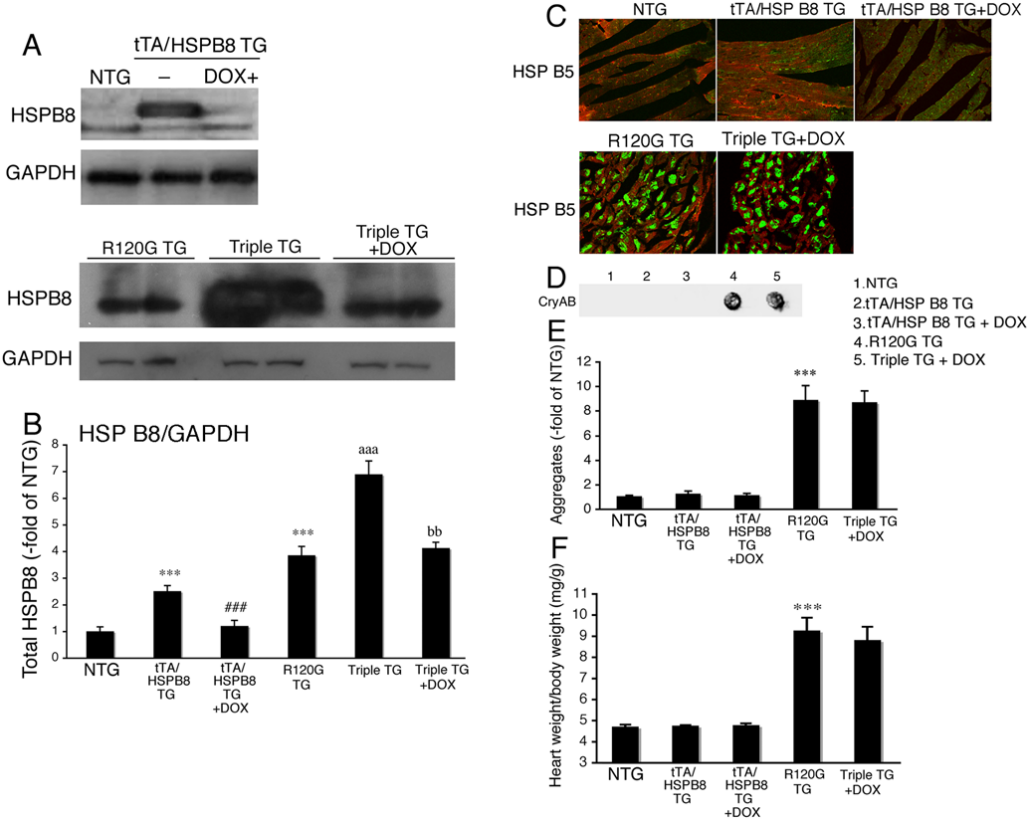
Effect of doxycycline (DOX) on aggregate formation and heart weight in tTA/HSP B8/R120G triple TG mice (Triple TG) at 30 weeks of age. DOX was administered via drinking water (1g/l) from 4 weeks of age. (A) Representative Western blots using hearts from tTA/HSPB8 double transgenic (tTA/HSPB8 TG) mice with DOX, HSPB5 R120G transgenic (R120G TG) and tTA/HSPB8/HSPB5 R120G triple transgenic (Triple TG) mice with DOX. (B) Quantitative analysis of HSPB8 levels (n = 4 mice). Values are the -fold increase relative to those in non-transgenic (NTG) mice whose values are arbitrarily set to 1. (C) Representative pictures of the immunohistochemistry are shown. Green color shows HSPB5 and red color shows troponin I. (D) Typical picture of the filter assay for the detection of the aggregates. The aggregates containing the mutant HSPB5 R120G protein are observed in R120G TG mice (No. 4) and triple TG mice with doxycycline (No. 5). (E) Quantitative analysis of aggregates (n = 6 mice). Values are the -fold increase relative to those in NTG mice (No.1) whose values are arbitrarily set to 1. (F) The ratios of heart weight per body weight (n = 6 mice). Cardiac enlargement is seen in R120G TG mice (No. 4) and triple TG mice (No. 5) whereas no difference is observed among NTG (No.1), tTA/HSPB8 TG (No. 2) or tTA/HSPB8 mice with DOX. *** *p*<0.001 vs. NTG mice; ### p<0.001 vs. tTA/HSPB8 TG mice; aaa p<0.001 vs. R120G TG mice; bb p<0.01 vs. Triple TG mice.

In another set of experiments, we treated tTA/HSPB8/R120G triple TG mice from 4 weeks to 30 weeks of age with doxycycline to shut down HSPB8 transgene expression. The levels of HSPB8 were similar between the tTA/HSPB8/R120G triple TG mice treated with doxycycline and the HSPB5 R120G TG mice (Figure 7A and B). The levels of aggregates as well as cardiac hypertrophy were similar between the tTA/HSPB8/R120G triple TG mice treated with doxycycline and the HSPB5 R120G TG mice (Figure 7C–F). These results imply that the overexpression of HSPB8 is critical to suppress the progression of cardiac disease in HSPB5 R120G TG mice.

### Inhibition of apoptotic cell death by GGA treatment or overexpression of HSPB8

**Figure 8:**
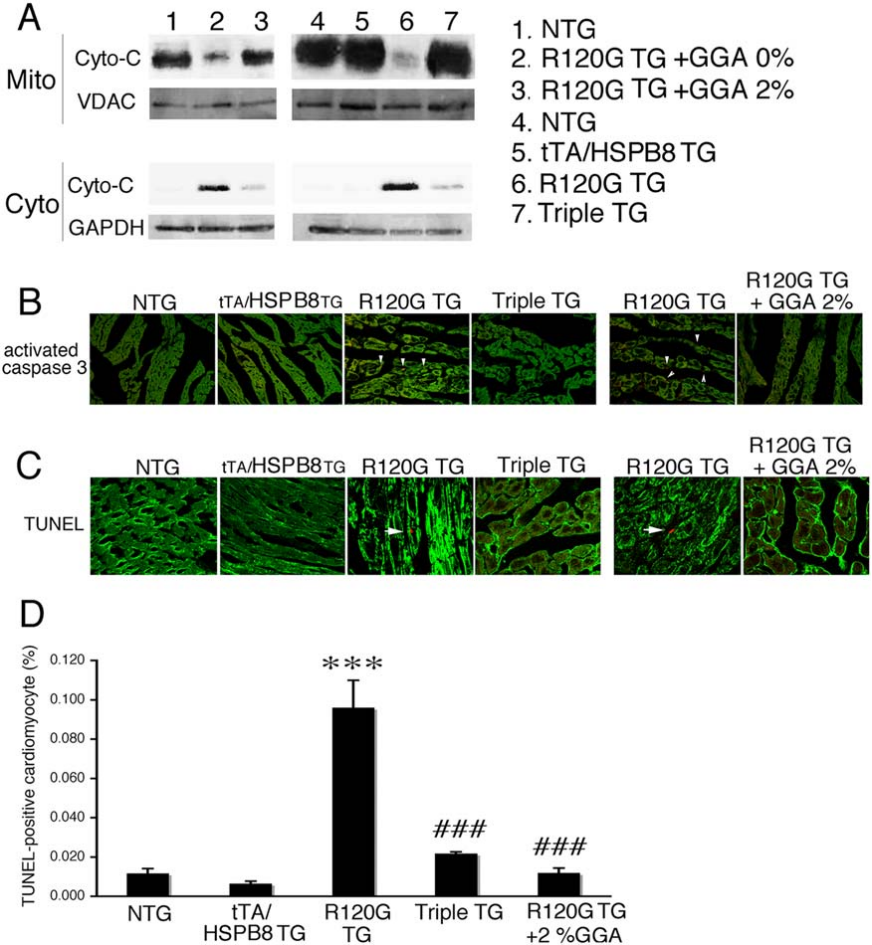
TUNEL-positive cardiomyocyte death in HSPB5 R120G TG (R120G TG) mice. (A) Cytochrome c was released from mitochondria (Mito) into the cytosolic fraction (Cyto) in R120G TG mice while GGA treatment (GGA 2%) and overexpression of HSPB8 inhibited its release. (B) Immunostaining for activated caspase-3 in sections derived from hearts at 30 weeks of age. Cardiomyocytes were identified by phalloidin staining for actin (green). Activated caspase-3 (arrowheads) is apparent in the HSPB5 R120G (R120G TG) section. GGA treatment (GGA 2%) and overexpression of HSPB8 (tTA/HSPB8/HSPB5 R120G triple TG; Triple TG) reduced caspase-3 activation. (C) TUNEL assays in hearts from HSPB5 R120G TG mice at 30 weeks of age. Cardiomyocytes were identified by phalloidin staining for actin (green). Positive signals were apparent only in the HSPB5 R120G-derived sections and are indicated by an arrow. GGA treatment (GGA 2%) and overexpression of HSPB8 (Triple TG) decreased the TUNEL-positive signals. (D) Quantification of TUNEL signals. Between 5×10^5^ nuclei were counted for each sample and the percentage of TUNEL-positive cells were determined (n = 4 mice).

We have shown that apoptotic cell death mediated through mitochondrial and caspase pathways plays a critical role in disease progression in HSPB5 R120G cardiomyopathy [18]. We hypothesized that GGA treatment and overexpression of HSPB8 mediate their effects through reduction in cell death. To test this hypothesis, we analyzed cytochrome c release from mitochondria, a process that can initiate the intrinsic apoptotic response. Cytochrome c was markedly decreased in the mitochondrial fraction of the R120G TG mouse heart (Figure 8A), and increased in the cytosol (Figure 8A) and activation of caspase-3 as well as increased TUNEL-positive cardiomyocyte death was detected in the R120G TG mouse hearts (Figure 8B–D). Both GGA treatment and overexpression of HSPB8 resulted in inhibition of cytochrome c release from mitochondria, decreased activation of caspase-3 and attenuated TUNEL-positive cardiomyocyte death in R120G TG mice (Figure 8A–D). These results suggest that GGA treatment and overexpression of HSPB8 inhibit apoptotic cell death through mitochondrial and caspase pathways.

## Discussion

In our previous study, we found that HSPB5 R120G, which can cause DRM, led to increased perinuclear aggresome formations that contain amyloid oligomer [3]. Continuous expression of the mutant HSPB5 R120G protein was required to sustain high concentrations of the amyloid oligomer, which correlated with depressed cardiac function as well as premature lethality [14]. It is hypothesized that amyloid oligomers can permeabilize cellular membranes and lipid bilayers, which may represent the primary toxic mechanism of amyloid pathogenesis [21]. Another previous study suggests that cellular toxicity induced by the amyloid oligomer is associated with mitochondrial function as well as induction of apoptotic cell death by cytochrome c release from mitochondria [18]. Thus, attenuation of amyloid oligomer formation might be an effective therapeutic strategy for the treatment of DRM. Our previous in vitro study showed that HSPB8 and HSPB1, which can bind to HSPB5 R120G, inhibited amyloid oligomer formation and reduced cell death in the R120G transfected cardiomyocytes [15]. In the present study, we extend these findings to the in vivo level.

Many studies using GGA focused on HSP70 induction [16], [20], [22], [23], [24]. In the present study, induction of HSP70 had already occurred in the HSPB5 R120G TG mouse hearts and only modest enhancement of HSP70 expression was observed with long-term GGA treatment. These results imply that although the effect of GGA on HSP70 induction is well characterized, it is not always the sole basis for the protective effect of GGA. Furthermore, overexpression of HSP70 failed to rescue the neurodegenerative disease and reduce aggregate formation in other amyloid-related neurodegenerative disease models that are caused by a polyglutamine repeat [25], [26], while GGA treatment was effective in mice with spinal and bulbar muscular atrophy (SBMA), which is also caused by a polyglutamine repeat [20]. Thus, HSP70 induction as a protective mechanism may be cell context dependent particularly as it relates to GGA treatment. In the model tested here, we found that long-term treatment with GGA results in HSPB8 induction and a similar level of overexpression of HSPB8 via transgenic manipulation recapitulates the protective effect of GGA in HSPB5 R120G cardiomyopathy. We have shown that HSPB8 can directly interact with HSPB5 R120G and inhibit amyloid oligomer and aggresome formation [15]. HSPB8 interacts with HSPB5 R120G and functions as a chaperone, assisting in the folding of the mutant protein [27], [28]. The present study confirms that induction of HSPB8 is beneficial for inhibiting DRM progression and that HSPB8 may play a critical role in mediating the protective effect of GGA.

HSP70 expression was significantly increased in the hearts of NTG mice treated with GGA, but there was no obvious induction in other small HSPs such as HSPB8, HSPB1 and HSPB5. In contrast to the NTG mouse hearts, small HSPs were induced in the HSPB5 R120G TG mouse hearts. Similar findings were observed in a spinal and bulbar muscular atrophy (SBMA) TG mouse model: oral administration of GGA up-regulated the expression of HSPs in the central nervous system in the SBMA TG mice while no induction was detected in NTG mice [20]. The exact mechanism(s) by which GGA treatment increases HSPB8 expression in HSPB5 R120G TG mice has not been fully elucidated. Since HSPs were already induced in both the HSPB5 R120G TG mouse hearts and the SBMA TG mouse central nervous systems, the effect of GGA on HSP induction may be heightened under conditions of stress. Further studies must address the effect of GGA on small HSP induction.

Many previous studies using GGA have analyzed the expression of HSP70 [16], [22], [23], [24]. GGA can directly bind to HSP70, and this binding results in dissociation between HSP70 and the HSF complex [24], [29], [30]. It is hypothesized that the dissociated HSF from HSP70 can predominately translocate to the nuclei and activate HSP transcription by binding HSF binding elements (HSE) in such targets as the HSP70 promoter. Another study suggests that activation of PKC (translocation of PKCdelta) by GGA plays a role in mediating HSF translocation [24]. Thus, these studies imply that the protein interaction between HSF and HSP70 is important for GGA-mediated HSP induction [24], [29], [30]. In mammalian cells, induction of HSP70 requires activation and nuclear localization of HSF-1 [30], [31]. These studies suggest that HSP70 may play a role in HSPB8 induction in the hearts of HSPB5 R120G TG mice. However, further enhancement of HSP70 induction by GGA treatment was modest in these hearts and HSF1 was already translocated into nuclei in the R120G TG mice in this study. Although transcriptional activity of the HSF1 is uncertain, depending on the location of HSF1 as assessed immunohistochemically, the HSP70-HSF interaction is not sufficient to explain GGA's effects completely. Some previous studies indicate that the protective effect of GGA may be explained by its modulation of nitric oxide [32], [33]. Another study indicates that mitochondria are targets for GGA-mediated protection against oxidative stress in human monocytes, independently of HSP70 [34]. These other mechanisms may be involved in the protective effects of GGA against DRM. Further studies are needed to elucidate the underlying mechanism of HSPB8 induction in DRM.
